# Favorable inhibitory effect of clodronate on hepatic steatosis in short bowel syndrome model rats

**DOI:** 10.1007/s00383-024-05858-y

**Published:** 2024-11-13

**Authors:** Yudai Tsuruno, Ayaka Nagano, Koshiro Sugita, Shun Onishi, Yumiko Tabata, Chihiro Kedoin, Masakazu Murakami, Keisuke Yano, Takafumi Kawano, Nao Hasuzawa, Masatoshi Nomura, Tatsuru Kaji, Yuko Bitoh, Satoshi Ieiri

**Affiliations:** 1 Department of Pediatric Surgery, Medical and Dental Area, Research and Education Assembly, Research Field in Medical and Health Sciences, Kagoshima University, 8-35-1, Sakuragaoka, Kagoshima, 890-8520 Japan; 2 Division of Pediatric Surgery, Department of Surgery, Kobe University Graduate School of Medicine, Kobe, Japan; 3 Division of Endocrinology and Metabolism, Department of Internal Medicine, Kurume University School of Medicine, Kurume, Japan; 4 Department of Pediatric Surgery, Kurume University School of Medicine, Kurume, Japan

**Keywords:** Short bowel syndrome, Intestinal failure-associated liver disease, Hepatic steatosis, Clodronate, VNUT inhibitor

## Abstract

**Purpose:**

This study investigated the anti-inflammatory effect of clodronate, a vesicular nucleotide transporter (VNUT) inhibitor, on intestinal-failure-associated liver disease (IFALD) in a rat model of short bowel syndrome (SBS).

**Methods:**

The rats underwent jugular vein catheterization for continuous total parenteral nutrition (TPN) and 90% small bowel resection. The animals were divided into the following groups: TPN/SBS (Control group), TPN/SBS/intravenous administration of low-dose clodronate (20 mg/kg twice per week; Low group), or TPN/SBS/intravenous administration of high-dose clodronate (60 mg/kg twice per week; High group). On day 7, the rats were euthanized. Hepatic steatosis and hepatocellular injury were also assessed.

**Results:**

Hepatic steatosis and lobular inflammation in the liver were observed in all groups. The High group showed histologically reduced hepatic steatosis compared with the Control group. IL-6 and Nlrp3 expression in the High group was significantly suppressed compared to that in the Control group. The expression of other inflammatory cytokines tended to be lower in the High dose group than in the control group. The lipid metabolism gene expression in the liver specimens showed no significant differences among the groups.

**Conclusion:**

The high-dose administration of clodronate may, therefore, inhibit hepatic steatosis and inflammation associated with IFALD in patients with SBS.

**Supplementary Information:**

The online version contains supplementary material available at 10.1007/s00383-024-05858-y.

## Introduction

Intestinal failure-associated liver function disorder (IFALD) is a serious complication in patients with short bowel syndrome (SBS). IFALD can cause biliary stasis, which can eventually lead to cirrhosis, particularly in newborns. IFALD is thought to be caused by a combination of factors including immaturity of the bile transport mechanism, excessive glucose administration due to total parental nutrition (TPN), and the inappropriate administration of fat emulsion [1, 2]. In IFALD, steatosis in the liver is similar to that observed in nonalcoholic fatty liver disease (NAFLD). Recently, the effect of clodronate on NAFLD has been reported [3]. Clodronate, a first-generation non-nitrogen bisphosphonate used to treat osteoporosis, is a potent vesicular nucleotide transporter (VNUT) inhibitor. VNUT is a protein involved in the secretion of intracellular ATP vesicles and it has been shown to be involved in all purine-signaling tissues. Clodronate has anti-inflammatory and osteoclast-suppressive effects. Purine signaling in the liver is closely related to the regulation of the glucose metabolism, lipid metabolism, inflammation, and the immune response [4]. We hypothesized that clodronate may be effective for IFALD because of the biliary stasis induced by inflammation and hepatic steatosis caused by various metabolic disorders.

We, therefore, investigated the anti-inflammatory effects of clodronate on IFALD in the SBS/TPN rat model in this study.

## Materials and methods

### Animals

Our experimental rats were 6-week-old male Sprague–Dawley (SD) rats weighing 180–220 g (Kyudo Co., Ltd., Saga, Japan). The rats were allowed to acclimatize themselves to the laboratory environment for 7 days before the experiments were started. During this period, the rats were housed individually in metabolic cages and all had ad libitum access to standard rat chow and water. The laboratory environment was maintained at a constant temperature of 23 ± 1 °C and humidity of 50 ± 10% with a 12-h light–dark cycle (lights on at 7:00 AM).

Following a period of environmental acclimation, the experimental rats underwent surgical procedures and then were subsequently maintained on TPN for 7 days. The rats were euthanized on the 7th postoperative day.

### Study design

The rats were randomly allocated into one of the three groups as follows: massive small bowel resection and TPN (SBS/TPN: Control group, *n* = 8); SBS/TPN with the intravenous administration of low-dose clodronate (Tokyo Chemical Industry Co., Ltd., Tokyo, Japan) (20 mg/kg/day; SBS/TPN/low-dose Clodronate: Low group, *n* = 8); SBS/TPN with the intravenous administration of high-dose clodronate (Tokyo Chemical Industry Co., Ltd., Tokyo, Japan) (60 mg/kg/day; SBS/TPN/high-dose Clodronate: High group, *n* = 8). The dosing regimen for clodronate was determined based on previous studies [5, 6]. Clodronate was administered as a single intravenous infusion via a central venous catheter on the day of surgery and then again on the 3rd postoperative day.

### Surgical procedures and maintenance methods

All rats were anesthetized with isoflurane (1.5% inhalation by mask) and underwent jugular vein catheterization using the cut-down method. The catheter was tunneled subcutaneously into the back and then connected to a swivel device. Subsequently, all rats underwent 90% small bowel resection, preserving 5 cm of the jejunum from the ligament of Treitz and 5 cm of ileum from the ileocecal valve. The proximal jejunum was anastomosed to the distal ileum end-to-end. Buprenorphine (0.01 mg/kg per dose subcutaneously) was administered for analgesia. The rats had ad libitum access to water after the surgery. TPN was delivered via a multichannel syringe pump (KDS Legato 200 Series Syringe Pump Series; KD Scientific, Inc., Holliston, MA, USA) using a regimen determined in our previous study. Cardiac puncture and exsanguination under general anesthesia were performed for euthanasia.

### Histological analysis

For the histological analysis of liver tissue, we performed a histological analysis of the liver specimens based on the NAFLD activity score, which was the degree of lipid accumulation (steatosis score) and the number of positive macrophages or T lymphocytes in ten randomly selected fields (lobular inflammation score).

### RNA extraction, reverse transcription and real-time polymerase chain reaction (PCR)

The PCR amplification was performed as described in our previous study [7]. The intestines were frozen and processed using TRIzol reagent (Thermo Fisher Scientific Inc.) to extract mRNA. The extracted RNA was then purified using a spin cartridge and quantified using a spectrophotometer. Complementary DNA (cDNA) was synthesized from purified RNA. Real-time PCR was performed to analyze the expression of the target genes using the QuantStudio 3 system (Thermo Fisher Scientific Inc.). The relative expression of the target genes was calculated using one of the control groups as a reference. The primers used in this study were purchased from Takara Bio Inc. All the primers used are listed in Supplementary Table S1.

### Serological analysis

The collected blood was immediately centrifuged at 1500 *g* for 15 min at 4 °C. All serum samples were stored at−  80 °C until use. The blood triglyceride and total cholesterol levels were measured using a point-of-care testing device (Spotchem EZ SP-4430; ARKRAY, Inc., Kyoto, Japan). The Spotchem II reagent was purchased from ARKRAY Inc.

### Statistical analyses

Statistical analyses were conducted using two-factor factorial ANOVA followed by Tukey’s post-hoc test. Data are presented as the mean ± standard error. Statistical significance was set at a *p*-value of < 0.05. All statistical analyses were performed using EZR (Saitama Medical Center, Jichi Medical University, Saitama, Japan), which is a graphical user interface for R (R Foundation for Statistical Computing, Vienna, Austria). More precisely, it is a modified version of R commander, which is designed to add statistical functions that are frequently used in biostatistics [8].

### Ethical approval

This experiment was conducted in accordance with the ARRIVE guidelines and is described in the text according to the checklist. All experimental procedures were approved by the Laboratory Animal Committees of Kagoshima University Graduate School of Medical and Dental Sciences and were performed in accordance with the “Guidelines for the Care and Use of Laboratory Animals” (approval number: MD23026).

## Results

### Histological findings of the liver specimens and a histological analysis based on the nonalcoholic fatty liver disease activity score

**Fig. 1:**
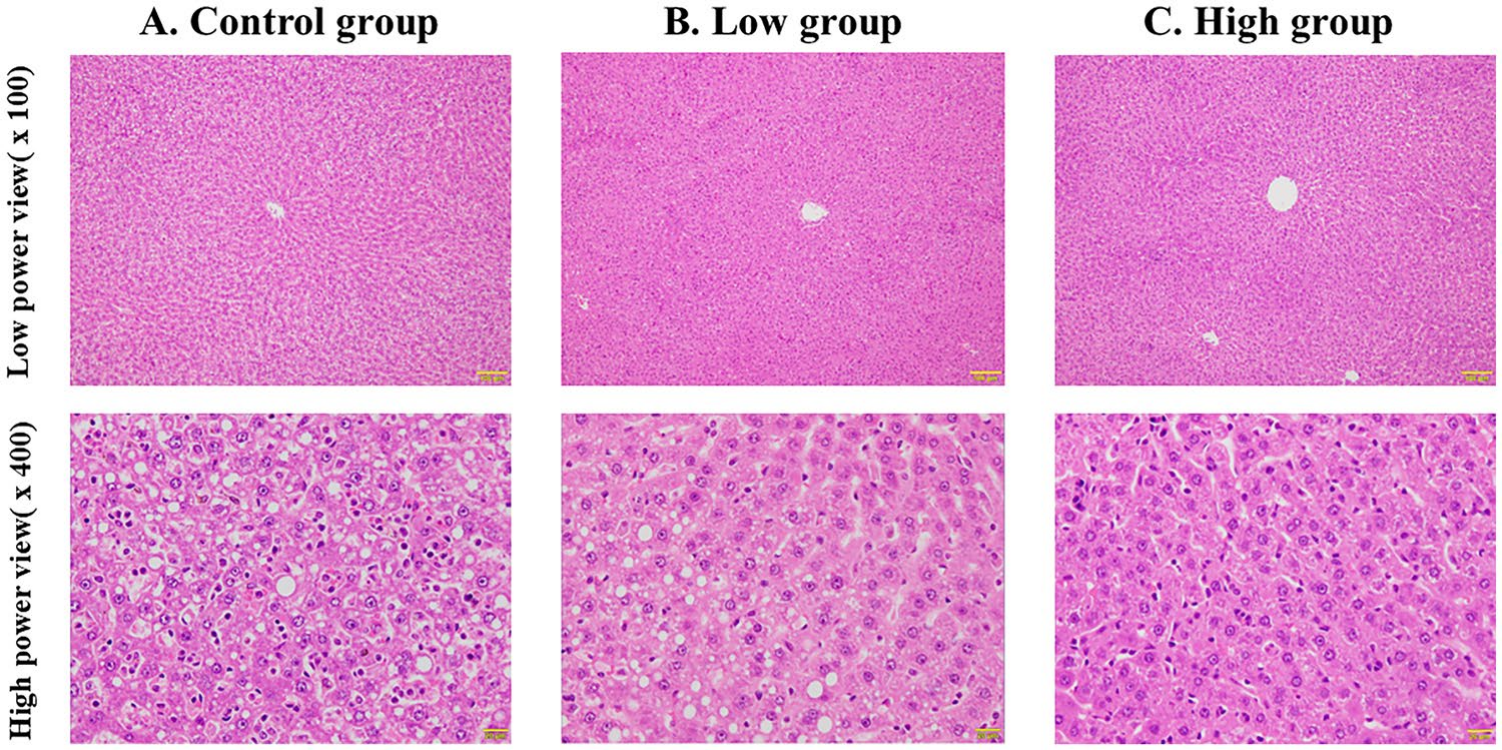
Histological findings of liver specimens. Hematoxylin–eosin staining; **A** Control group; **B** Low group; **C** High group. Top row: low-power view (× 100); bottom row: high-power view (× 400)

**Fig. 2:**
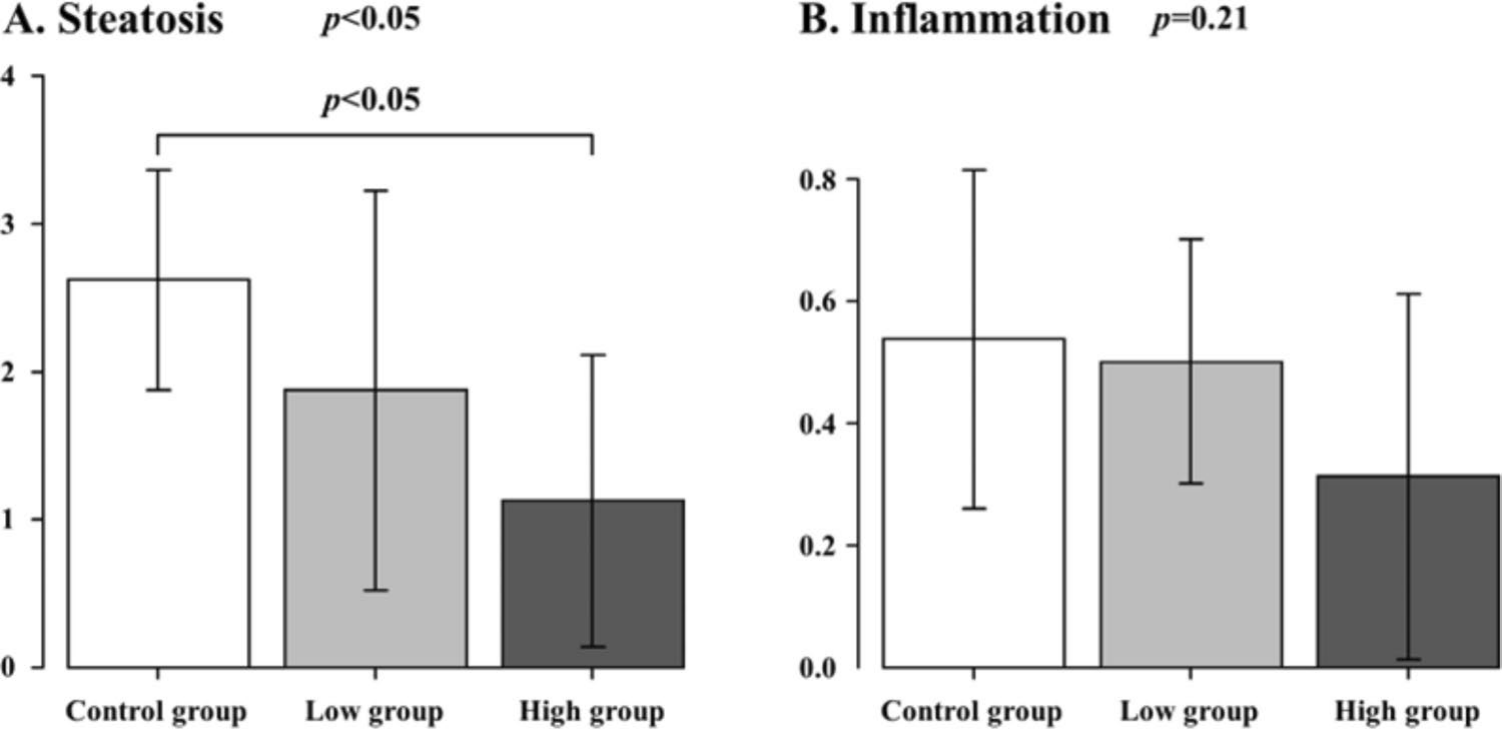
Histological analysis based on the nonalcoholic fatty liver disease activity score. Control group vs. Low group vs. High group. **A** Steatosis, **B** Inflammation

The histological findings of hematoxylin–eosin staining are shown in Fig. 1. Hepatic steatosis and inflammatory cell infiltration were observed in all the groups. A histological analysis based on the NAFLD activity score is shown in Fig. 2. The High group significantly showed the lowest hepatic steatosis score among all the groups (Control group vs. Low group vs. High group: 2.6 ± 0.7 vs. 1.9 ± 1.4 vs. 1.1 ± 1.0, *p* = 0.03). Inflammatory cell filtration was not significantly different among all the groups, but the High group showed the lowest score (Control group vs. Low group vs. High group: 0.5 ± 0.3 vs. 0.5 ± 0.2 vs. 0.3 ± 0.3, *p* = 0.21).

### Inflammation-related gene expressions in the liver specimens

**Fig. 3:**
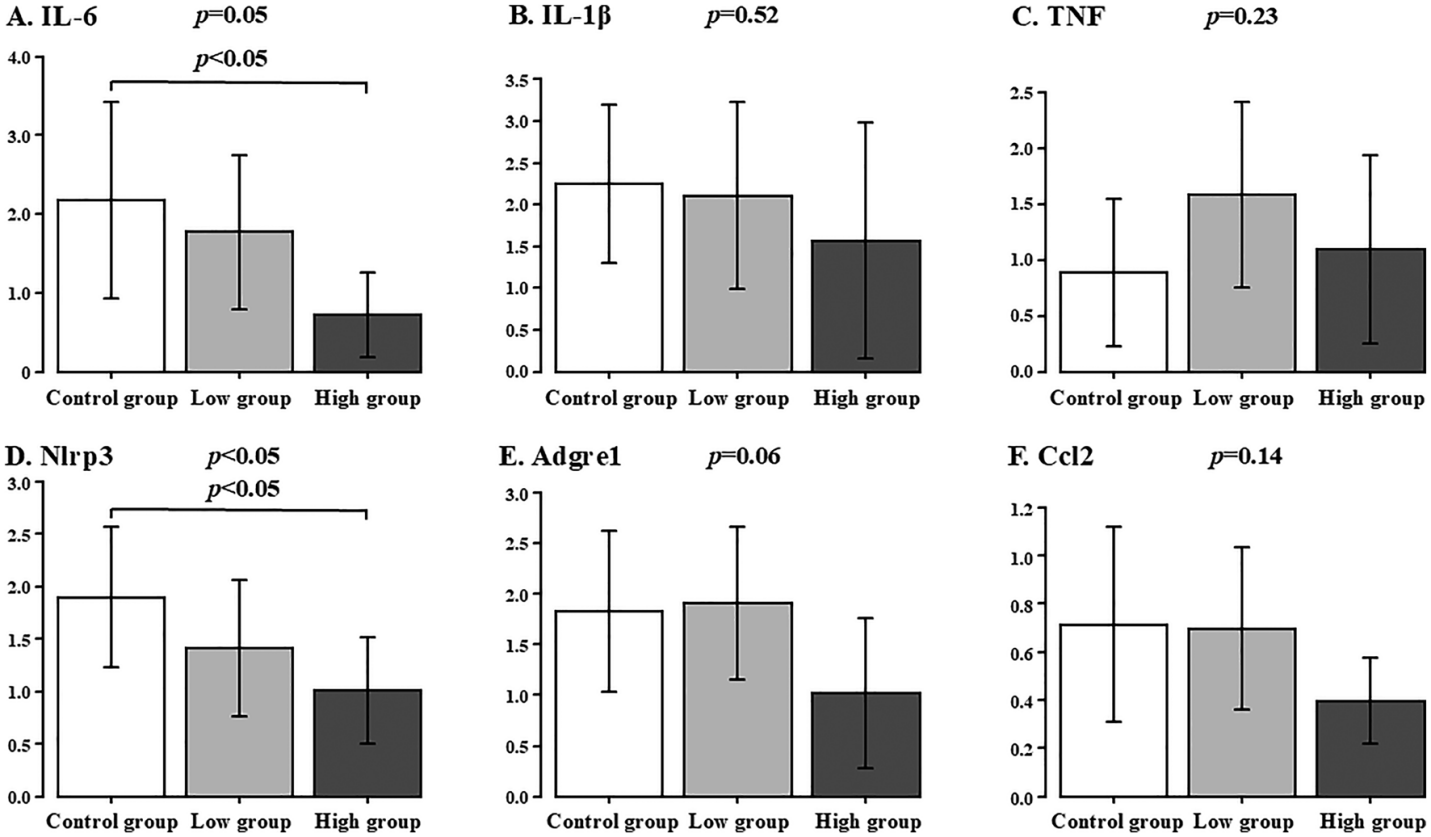
Gene Expressions of inflammation factors in the liver specimens. Control group vs. Low group vs. High group. **A** IL-6, **B** IL-1β, **C** TNF, **D** Nlrp3, **E** Adgre1, **F** Ccl2

The inflammation-related gene expression in the liver specimens is shown in Fig. 3. The IL-6 expression did not show any significant difference among all the groups, but the expression tended to be lower in the High group (Control group vs. Low group vs. High group: 2.2 ± 1.2 vs. 1.8 ± 1.0 vs. 0.7 ± 0.5, *p* = 0.05). The IL-1β expression did not show any significant difference among all the groups (Control group vs. Low group vs. High group: 2.2 ± 0.9 vs. 2.1 ± 1.1 vs. 1.6 ± 1.4, *p* = 0.52). The TNF expression did not show any significant difference among all the groups (Control group vs. Low group vs. High group: 0.9 ± 0.7 vs. 1.6 ± 0.8 vs. 1.1 ± 0.8, *p* = 0.23).

The Nlrp3 expression in the High group significantly showed the lowest score among all groups (Control group vs. Low group vs. High group: 1.9 ± 0.7 vs. 1.4 ± 0.6 vs. 1.0 ± 0.5, *p* = 0.04). The Adgre1 expression did not show any significant difference among all the groups, but the expression tended to be lower in the High group (Control group vs. Low group vs. High group: 1.8 ± 0.8 vs. 1.9 ± 0.8 vs. 1.0 ± 0.7, *p* = 0.06). The Ccl2 expression did not show any significant difference among all the groups (Control group vs. Low group vs. High group: 0.7 ± 0.4 vs. 0.7 ± 0.3 vs. 0.4 ± 0.2, *p* = 0.14).

### Lipid metabolism-related gene expressions in the liver specimens

**Fig. 4:**
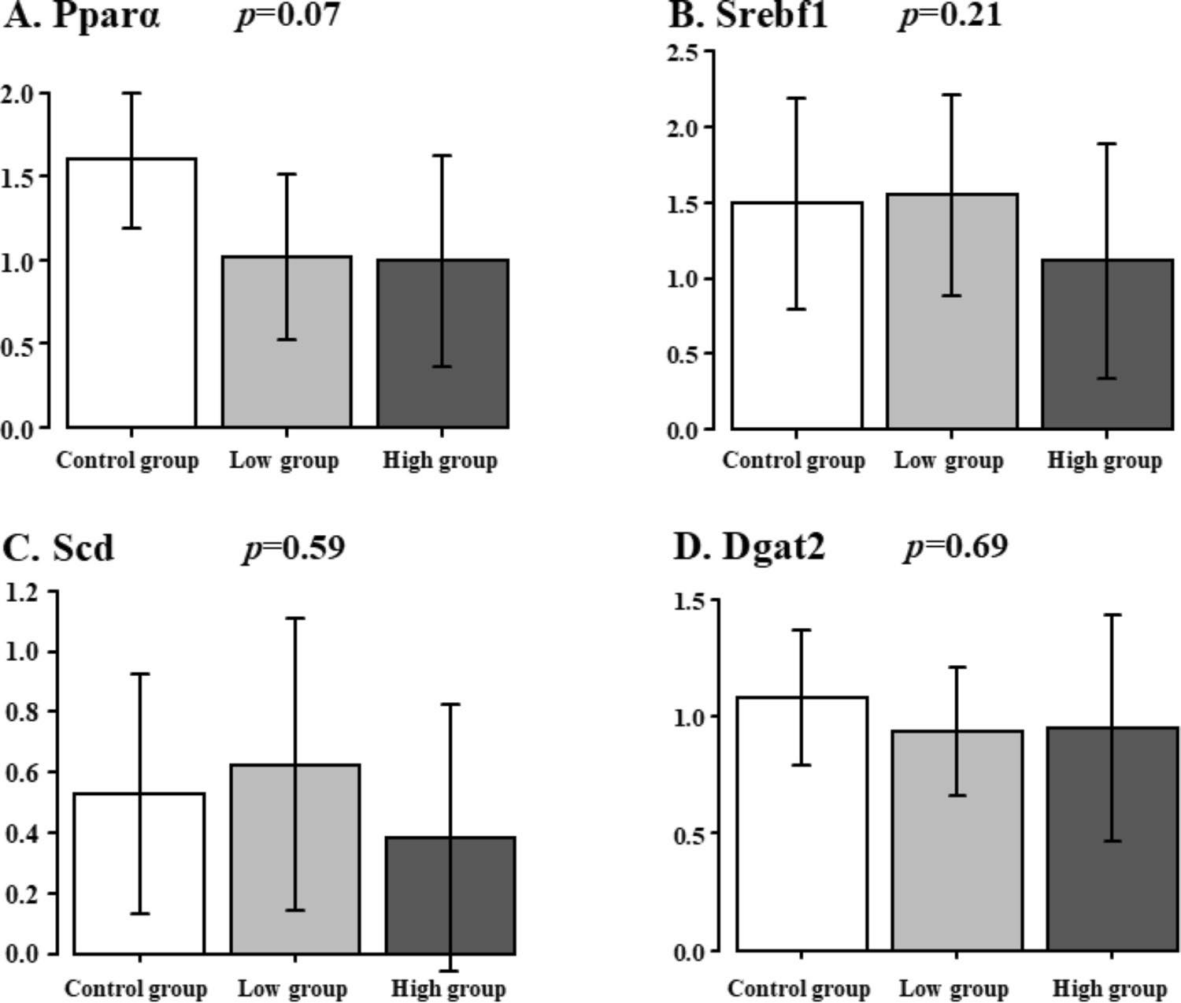
Lipid metabolism-related gene expressions in the liver specimens. Control group vs. Low group vs. High group. **A** Pparα, **B** Srebpf1, **C** Scd, **D** Dgat2

The lipid metabolism-related gene expression in the liver specimens is shown in Fig. 4. The Pparα expression did not show any significant difference among all the groups, but the expression tended to be higher in the Control group (Control group vs. Low group vs. High group: 1.6 ± 0.4 vs. 1.0 ± 0.5 vs. 1.0 ± 0.6, *p* = 0.07). The Srebf1 expression did not show any significant difference among all the groups (Control group vs. Low group vs. High group: 1.3 ± 0.4 vs. 1.2 ± 0.3 vs. 0.8 ± 0.7, *p* = 0.21). The Scd1 expression did not show any significant difference among all the groups (Control group vs. Low group vs. High group: 0.5 ± 0.4 vs. 0.6 ± 0.5 vs. 0.3 ± 0.4, *p* = 0.59). The Dgat2 expression did not show any significant difference among all the groups (Control group vs. Low group vs. High group: 1.1 ± 0.3 vs. 0.9 ± 0.3 vs. 0.9 ± 0.5, *p* = 0.69).

### Serum levels of total cholesterol and triglycerides

**Fig. 5:**
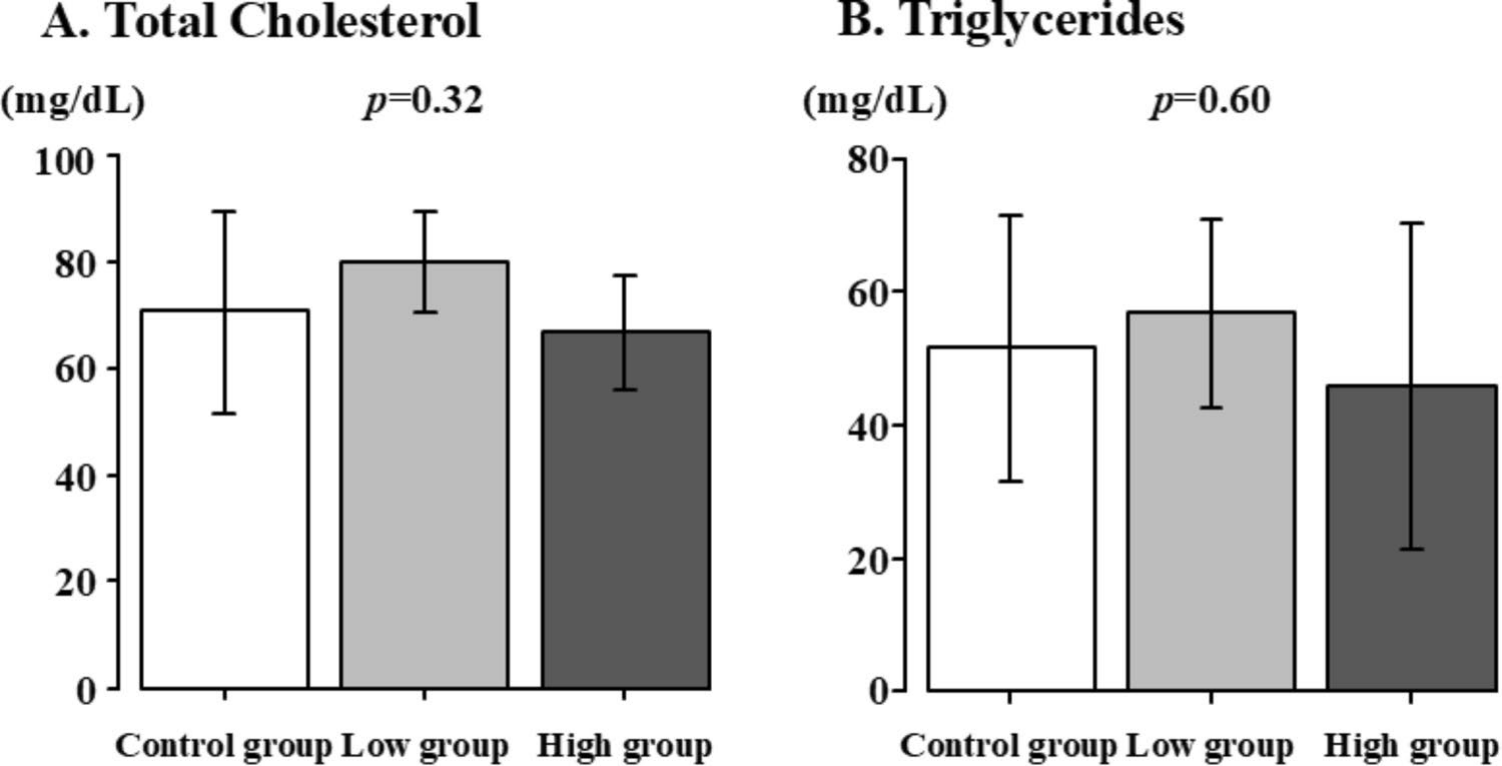
Serum levels of total cholesterol and triglycerides. Control group vs. Low group vs. High group. **A** Total Cholesterol, **B** Triglycerides

The serum levels of total cholesterol and triglycerides are shown in Fig. 5. Total cholesterol (Control group vs. Low group vs. High group: 70.7 ± 18.9 vs. 80.2 ± 9.5 vs. 66.8 ± 10.8, *p* = 0.32) and triglycerides (Control group vs. Low group vs. High group: 51.5 ± 19.9 vs. 56.7 ± 14.2 vs. 45.7 ± 24.6, *p* = 0.60) did not show any significant difference among all the groups.

### Hepatic fibrosis-related gene expressions in the liver specimens

**Fig. 6:**
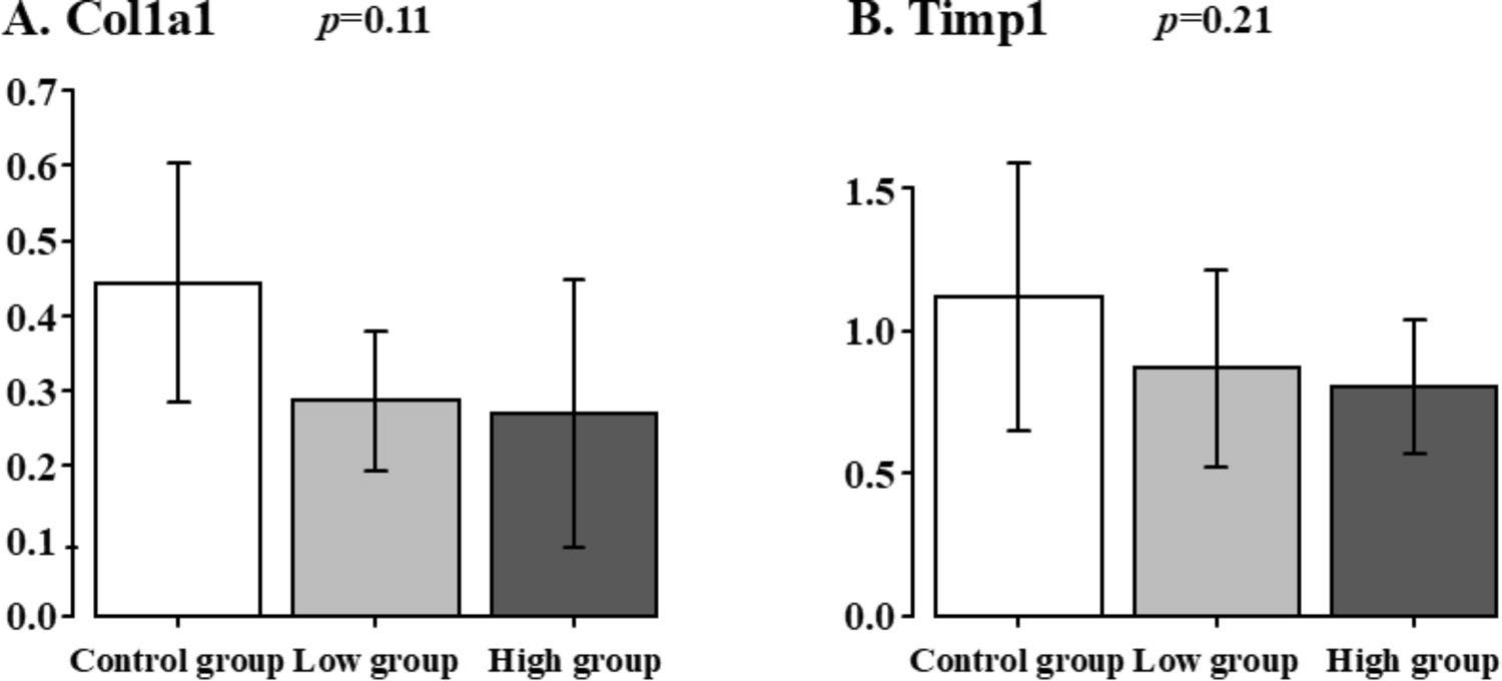
Hepatic fibrosis-related gene expressions in the liver specimens. Control group vs. Low group vs. High group. **A** Col1a1, **B** Timp1

The Hepatic fibrosis-related gene expression in the liver specimens is shown in Fig. 6. The Col1a1 expression did not show any significant difference among all the groups (Control group. Low group vs. High group: 0.4 ± 0.2 vs. 0.3 ± 0.1 vs. 0.3 ± 0.2, *p* = 0.11). The Timp1 expression did not show any significant difference among all the groups (Control group vs. Low group vs. High group: 1.1 ± 0.5 vs. 0.9 ± 0.3 vs. 0.8 ± 0.2, *p* = 0.21).

## Discussion

The major findings of this study are as follows: (1) In the Control group of the SBS/TPN rat model, hepatic steatosis and lobular inflammation in the liver were observed at 1 week after surgery. (2) While the Low and Control groups showed no histological differences in liver steatosis, the High group showed histologically reduced hepatic steatosis compared to that of the Control group. (3) The IL-6 levels in the liver at 1 week after surgery were also suppressed in the High group compared to the control group. (4) The expression of Nlrp3 inflammasome was suppressed in the High group compared to that in the Control group. The expression of other inflammatory cytokines tended to be lower in the High group than in the Control group. (5) The gene expression of lipid metabolism in the liver specimens showed no significant differences among all the groups.

The main pathogenesis of IFALD involves biliary stasis and hepatocellular damage induced by inflammation and hepatic steatosis due to metabolic disorders of various causes. Nlrp3 is an inflammasome that induces inflammatory responses via pathogen-associated molecular pattern or danger-associated molecular pattern (DAMPs). The abnormal activation of Nlrp3 has been reported to be associated with several inflammatory diseases such as diabetes and Alzheimer’s disease [9]. DAMPs and ATP are released from parenchymal cell damage during acute liver injury and inflammatory macrophages are activated. As a result, hepatocytes are damaged and produce tissue inhibitors of metalloproteinases (TIMPs). IL-6 is an inflammatory cytokine that has a variety of functions and stimulates cells in multiple pathways involved in acute inflammation [10]. The lower levels of Nlrp3 and IL-6 in the High group may be due to the anti-inflammatory effects of clodronate related to purine signaling. IL-1β is a pro-inflammatory cytokine that causes tissue damage and it is produced mainly by monocytes and macrophages via the Nlrp3 inflammasome. On the other hand, IL-1β is also produced by proteases of neutrophil, and thus, under neutrophil-infiltrating inflammatory conditions, IL-1β is activated extracellularly without inflammasome. Therefore, the fact that IL-1β was not significantly lower in the High group may be due to the activation of IL-1β by another pathway that is not via Nlrp3 inflammasome [11, 12].

In NAFLD, a high-fat diet and hyperinsulinemia cause hepatic steatosis, which eventually progresses to NASH. The IFALD rat model of SBS/TPN, hyperinsulinemia and hyperlipidemia due to TPN-induced hyperglycemia and surgical invasion are thought to cause hepatic steatosis. Srebf1 regulates the transcription of the genes that regulate fatty acid and triglyceride synthesis. Srebf1 overexpression is strongly associated with insulin resistance, diabetes, and NAFLD [13–16]. Pparα agonists have also been reported to inhibit Srebf1 expression [17, 18] and Pparα activation reduces the development of hepatic steatosis [19]. The Srebf1 expression in the High group tended to be lower than that in the Control group; however, the Pparα expression also tended to be lower. Other metabolic regulators related to fat metabolism did not decrease significantly in the High and Low groups. The discrepant findings between SREBF1 and PPARα suggest that clodronate had no impact on this signaling pathway in our study. SREBF1 is also a downstream target of the insulin receptor. Our study has not yet fully elucidated the signaling pathway from the insulin receptor to SREBF1. Therefore, further investigation is required to understand the detailed mechanism. While we did not observe direct inhibition of the TG metabolic pathway, we hypothesize that improved insulin sensitivity could be a potential underlying mechanism. Some NAFLD cases progress to NASH with progressive inflammation and fibrosis. Tatsushima et al. reported that inflammation and fibrosis were markedly suppressed in VNUT knockout mice in a high-fat diet-induced NASH mouse model [20]. In this study, there was no difference in the expression levels of Col1a1 and Timp1, which are associated with liver fibrosis, among all the groups. Pathologically, there were no findings of liver fibrosis in any of the groups. These results suggest that the SBS/TPN rat model represents the acute phase of IFALD, in which fibrosis has not yet occurred.

While ω-3 fatty acids, which target the arachidonic acid cascade related to fatty acids, are clinically used for the treatment of inflammation in patients with IFALD, the results of this study highlight the potential of anti-inflammatory drugs targeting immune cells to suppress hepatic steatosis without significantly affecting metabolism. Future research focusing on inflammation in IFALD may contribute to elucidating the pathogenesis of IFALD and developing novel therapeutic agents. To comprehensively investigate the pathogenesis of IFALD, animal models that accurately mimic the complex clinical presentation, including sepsis and cholestasis, are required. The inflammatory processes within these models need to be thoroughly characterized.

### Limitations

Although there was some scattering of the data, we refrained from using more animals than we had previously prepared for this experiment due to animal ethical reasons. Due to the limited number of animals, our statistical interpretation was, therefore, limited.

## Conclusions

The high-dose administration of clodronate may thus inhibit hepatic steatosis and inflammation in SBS. Further detailed studies on the mechanism of hepatic steatosis inhibition are, therefore, required.

## Supplementary Information

Below is the link to the electronic supplementary material.

Supplementary file1 (DOCX 17 KB)

## Abbreviations

VNUT: Vesicular nucleotide transporter
IFALD: Intestinal failure-associated liver disease
TPN: Total parenteral nutrition
SBS: Short bowel syndrome
NAFLD: Nonalcoholic fatty liver diseases
ATP: Adenosine triphosphate
IL6: Interleukin-6
IL-1β: Interleukin-1β
TNF: Tumor necrosis factor
SD rats: Sprague–Dawley rats
PCR: Polymerase chain reaction
RNA: Ribo nucleic acid
DNA: Deoxyribo nucleic acid
NASH: Nonalcoholic steatohepatitis
Pparα: Peroxisome proliferator-activated receptor
Srebf1: Sterol regulatory element bindings transcription factor 1

## Declarations

### Data availability

The datasets generated and/or analyzed during the current study are available from the corresponding author upon reasonable request.
