# Supplementary material for: Favorable inhibitory effect of clodronate on hepatic steatosis in short bowel syndrome model rats
Source: Pediatr Surg Int. 2024 Nov 13;40(1):307. doi: 10.1007/s00383-024-05858-y (PMC11561034; doi:10.1007/s00383-024-05858-y)
Supplement: Supplementary file 1 — Supplementary file1 (DOCX 17 KB) [file 383_2024_5858_MOESM1_ESM.docx]

**Supplementary Table 1. Primer for polymerase chain reaction**

| **Target** | **Forward** | **Reverse** |
| --- | --- | --- |
| **PPIA (KEEPING)** | 5’-GGCAAATGCTGGACCAAACAC-3’ | 5’-AAACGCTCCATGGCTTCCAC-3’ |
| **Nlpr3** | 5’-GCGTGGGACTGAAGCATCTG | 5’-AACCAATGCGAGATCCTGACAA |
| **Adgre1** | 5’-ACGGAGACAGGATTCATTTGGAG | 5’-ACCCACAGTGTCCAGGCAAG |
| **Ccl2** | 5’-CTATGCAGGTCTCTGTCACGCTTC | 5’-CAGCCGACTCATTGGGATCA |
| **IL-6** | 5’-TTGTATGAACAGCGATGATGCAC | 5’-CCAGGTAGAAACGGAACTCCAG |
| **IL-1β** | 5’-GGCAACTGTCCCTGAACTCAAC | 5’-CATCTGGACAGCCCAAGTCAA |
| **TNF** | 5’-CTCCGGGCTCAGAATTTCCA | 5’-ATCGACATTCCGGGATCCAG |
| **Pparα** | 5’-ACGCATGTGAAGGCTGCAA | 5’-CTTGTGGAAACGGCAGTACTGG |
| **Slebf1** | 5’-CGGGACAGCTTAGCCTCTACATC | 5’-GGCTGAAGCTGCTGACTGTTG |
| **Scd** | 5’-GCGTACAGTGCCAAATGCTCA | 5’-GGCATTTCCAGAGGTTTCCAG |
| **Dgat2** | 5’-GGAACCGCAAAGGCTTTGTAA | 5’-GGGCGAAACCAATATACTTCTGGA |
| **Col1a1** | 5’-GGGCAAGACAGTCATCGAATACAA | 5’-CCAATGTCCATTCCGAATTCC |
| **Timp1** | 5’-GCCTTCGTAAAGACCTATAGTGCTG | 5’-CCATGAGGATCTGATCTGTCCA |
